# Identification of severe fever with thrombocytopenia syndrome virus genotypes in patients and ticks in Liaoning Province, China

**DOI:** 10.1186/s13071-022-05237-3

**Published:** 2022-04-04

**Authors:** Xiao-Hu Han, Yue Ma, Hong-Yan Liu, Dan Li, Yan Wang, Feng-Hua Jiang, Qing-Tian Gao, Feng Jiang, Bao-Shan Liu, Guo-Shun Shen, Ze-Liang Chen

**Affiliations:** 1 Key Laboratory of Livestock Infectious Diseases, Ministry of Education, Shenyang Agricultural University, Shenyang, Liaoning Province 110866 People’s Republic of China; 2 The Sixth People’s Hospital of Shenyang, Shenyang, Liaoning Province 110866 People’s Republic of China; 3 Dandong Service Center of Agricultural and Rural Development, Dandong, Liaoning Province 118000 People’s Republic of China

**Keywords:** Severe fever with thrombocytopenia syndrome, Phlebovirus, Ticks, Genotype

## Abstract

**Background:**

Severe fever with thrombocytopenia syndrome (SFTS), caused by the SFTS virus (SFTSV), is an acute infectious disease transmitted by ticks that has recently been identified. There are no reports of epidemic serotypes in Liaoning Province, PR China. The aim of this study was, therefore, to identify genotypes of SFTSV in this province.

**Methods:**

In 2019, quantitative PCR testing was performed on 17 patients suspected of being infected with SFTS in Liaoning Province and on 492 ticks from the counties and cities surrounding the patients’ residences. Four samples were subjected to virus isolation and whole-genome amplification.

**Results:**

Molecular diagnostic results confirmed SFTSV infection in five of the 17 suspected cases of SFTS and in 12 of the 492 ticks, with a prevalence of 2.4%. Four strains of SFTSV were successfully isolated from patients’ blood and ticks. Phylogenetic analysis after whole-genome amplification and sequencing showed that they all belonged to genotype A of SFTSV.

**Conclusions:**

This study is the first to determine the genotype of SFTSV in patients and ticks in Liaoning Province, PR China. The results deepen our understanding of the SFTS epidemic and provide information on the variability in mortality rate among genotypes.

**Graphical Abstract:**

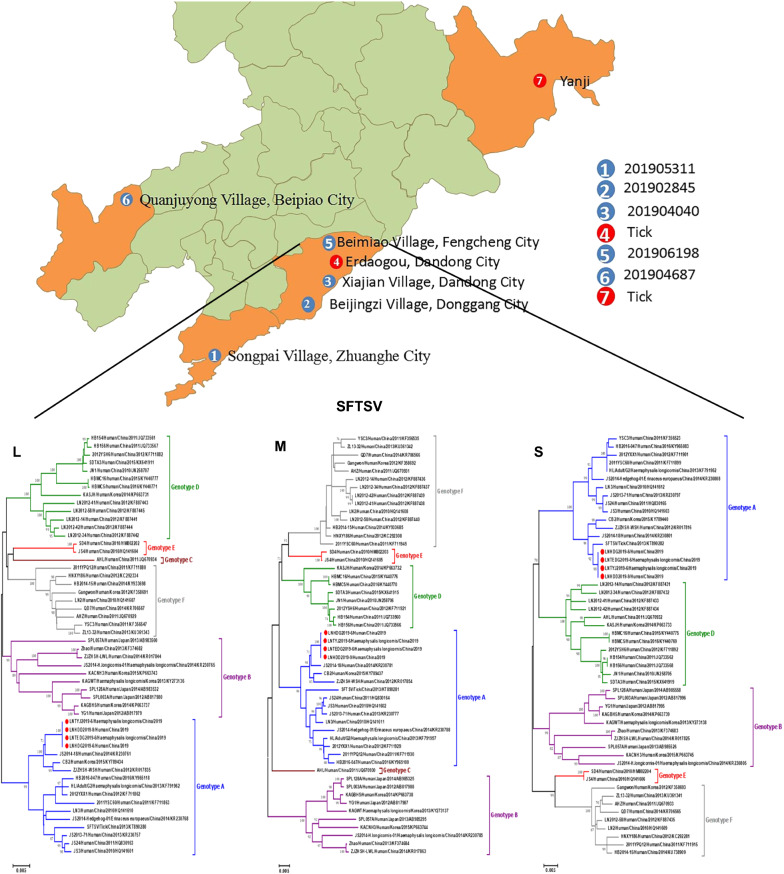

**Supplementary Information:**

The online version contains supplementary material available at 10.1186/s13071-022-05237-3.

## Background

Severe fever with thrombocytopenia syndrome (SFTS) is a new acute infectious disease that was first reported in 2009 in rural areas of the Hubei and Henan provinces in PR China [1]. The pathogen of the disease was initially classified as genus *Phlebovirus* [1], but in 2020 it was designated as *Dabie bandavirus* (genus *Bandavirus*; family *Phenuiviridae*) by the International Committee on Taxonomy of Viruses (ICTV). The main clinical manifestations of SFTS are fever, anorexia, muscle pain, chills, lymphadenopathy, leukopenia, thrombocytopenia and multiple organ failure [2, 3]. Severe cases can even lead to death.

SFTS virus (SFTSV) is an arbovirus mainly carried by ticks [4].* Haemaphysalis longicornis*,* Amblyomma testudinarium*,* Ixodes nipponensis* and* Rhipicephalus microplus* are the four main tick species that cause infections [5]. It can also be transmitted from person to person by direct contact with the blood or mucus of an infected person [6–8]. The general population is susceptible; therefore, its epidemic area tends to expand following initial infection [5]. Thousands of incidents of SFTS have been reported in more than 16 provinces and cities in China [9] as well as in the Republic of Korea [10], Japan [11] and the USA [12].

Liaoning Province is located in northeast China and borders North Korea, Russia, Japan and South Korea. Its eastern territory is mountainous and inhabited by ticks. SFTS is prevalent in Liaoning Province, with 183 cases confirmed in 2011–2014 [9]. Its prevalence [13], symptoms [2, 14] and trends [15] in Liaoning Province have been studied, but so far there are no reports of pathogen isolation and dominant epidemic strains.

The aim of this study was to identify genotypes of SFTSV in Liaoning Province. We therefore attempted to isolate the SFTS pathogen from the blood of individuals with suspected SFTS and from vector ticks. Our findings provide insights into the prevalence of SFTSV in Liaoning Province, as well as its differentiation.

## Methods

### Specimen collection

**Fig. 1:**
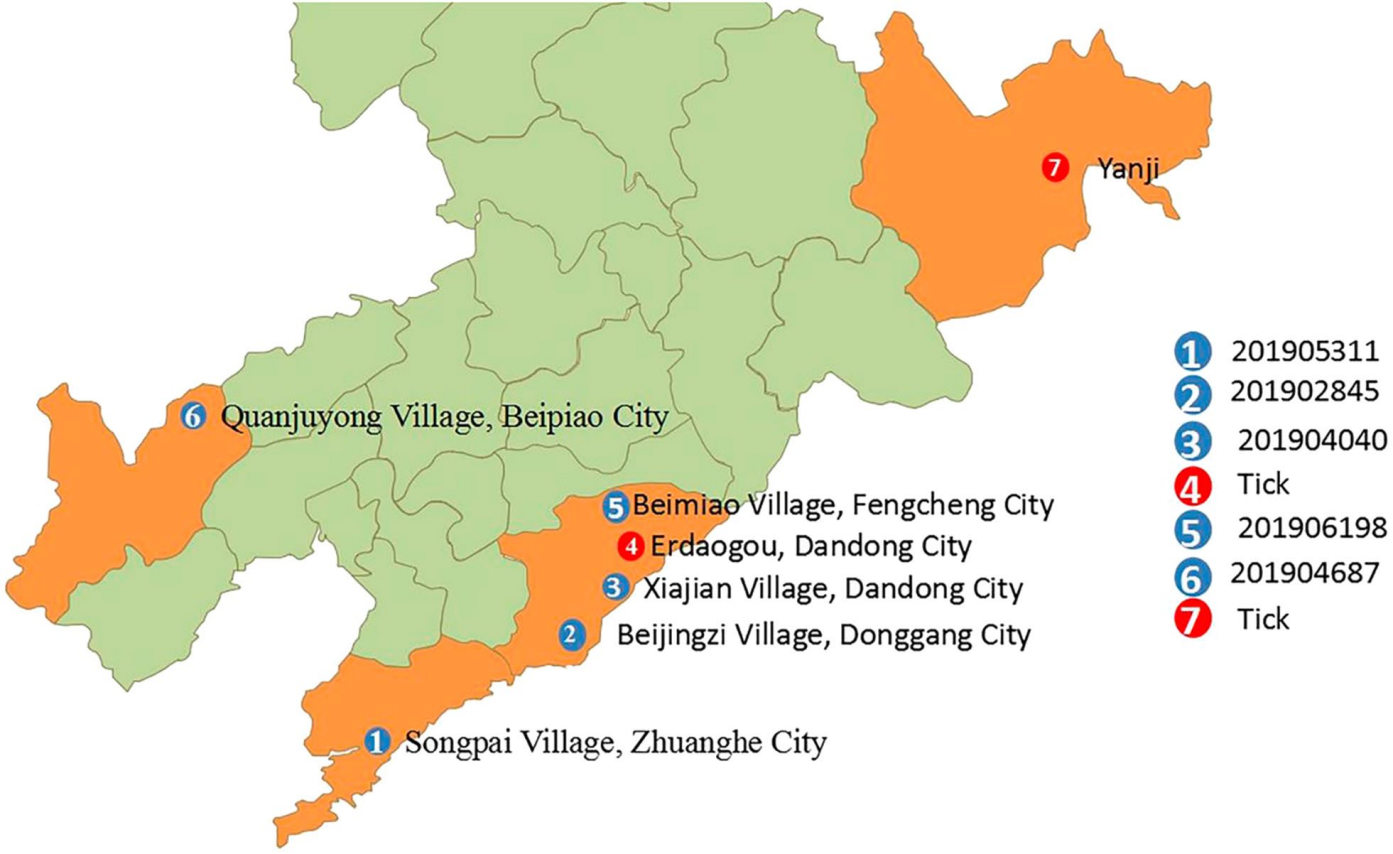
Region of patients and ticks in the study. Number coding:* 1‒6* locations where samples were taken from 17 patients,* 1‒7* locations where ticks were collected,* 4, 7* locations were SFTSV was isolated from ticks. SFTSV, Severe fever with thrombocytopenia syndrome

Between May and October 2019, 17 suspected cases of SFTS were reported by the Shenyang Infectious Disease Hospital, Shenyang, Liaoning Province. All 17 patients exhibited symptoms and were farmers, mainly from forest areas, who had been in contact with animals (rats) or been bitten by tick(s). Serum was collected from these patients and stored at − 80 °C until testing. A total of 492 adult tick samples (not collected in Beipiao City) were collected from patients’ residences and surrounding areas, including Dandong and Dalian cities, which are in forest areas of the Changbai Mountain range extending along the border area between China and North Korea (Fig. 1). After morphological identification, the tick samples were ground in liquid nitrogen. PBS was then added followed by centrifugation at 10,000 *g* for 10 min as preparation for nucleic acid extraction of the suspension.

### Nucleic acid extraction

Total nucleic acids were extracted from the sera of the 17 patients suspected of having SFTS and from the tick suspensions, using the EasyPure Viral DNA/RNA Kit (TransGen Biotech, Beijing, China) according to the manufacturer’s instructions.

### Tick species identification

Tick species were first identified based on morphological characters, and then the* 16S* ribosomal (rRNA) of partial samples was amplified and sequenced following a previously reported method [16]. For a detailed analysis of* Haemaphysalis*, we analyzed the extended sequence by PCR-restriction fragment length polymorphism (RFLP) of the* 16S* rRNA gene [17].

### SFTSV detection

SFTSV was detected using the following primer pairs (560 bp) targeting the M gene [18]: MF3 (5′-GATGAGATGGTCCATGCTGATTCT-3′) and MR2 (5′-CTCATGGGGTGGAATGTCCTCAC-3′). Reverse transcription and PCR amplification were performed using the HiScript II Q RT SuperMix for quantitative (q)PCR (+ gDNA wiper; Vazyme Biotech, Nanjing, China), according to the manufacturer's instructions, after DNase treatment of total nucleic acids. PCR conditions were: pre-denaturation for 5 min at 94 °C, followed by 40 cycles of 94 °C for 30 s, 55 °C for 30 s and 72 °C for 1 min.

### Virus isolation

Tick suspensions and patient sera that were determined positive for SFTSV by RT-PCR [19] were filtered through a 0.22 µm bacterial filter and mixed with the cell maintenance solution (Dulbecco's modified Eagle medium containing 2% foetal bovine serum). The mixture was added to the Vero cell layer and cultured at 37 °C under 5% CO_2_. Cytopathic effects (CPEs) were observed daily. Cells exhibiting CPEs were collected and verified by RT-PCR to verify successful infection. The infected cells were stored at − 80 °C until analysis.

### SFTSV whole-genome amplification

The SFTSV genome is composed of three independent gene segments: L segment (6368 bp; encodes the RNA-dependent RNA polymerase), M segment (3378 bp; encodes the membrane protein precursor), and the antisense S segment (1744 bp; encodes the non-structural S protein and nucleocapsid protein) [20]. Primers that were previously designed for the amplification of S (three primer sets), M (seven primer sets), and L (13 primer sets) segments were used for whole-genome sequencing [21] (Table 1). Amplification was performed on a thermal cycler at 94 °C for 30 s, 55 °C (63 °C) for 30 s, and 72 °C for 1 min for 35 cycles.

**Table 1:** SFTSV genome sequencing primer list

Primer name	Primer sequence 5′ → 3′	Length (bp)	Tm (°C)	Start-stop	Amplicon (bp)
SF1	TACACAAAGACCCCCTTCATTTGG	24	61.29	1–24	749
SR1	GCTCATCATCCTCATCCAAGACAC	24	61.28	727–750	
SF2	CTCCTCCAGATAGAGTCACTTGCA	24	61.41	582–605	646
SR2	TGAAGCCACAACCAAGACCCT	21	61.88	1207–1227	
SF3	TAAACTTCTGTCTTGCTGGCTCC	23	60.81	1123–1145	623
SR3	ACACAAAGAACCCCAAAAAAGG	23	57.65	1724–1745	
MF1	ACACAGTAGACGGCCAACAATGAT	24	62.61	1–24	587
MR1	TCTCCTCAGGGATGGGTGTCA	21	62.04	567–587	
MF2	GATAGTTCCTGGGCCTTCATACAA	24	60.14	370–393	640
MR2	GAAACCRTAGCACTTTGGTCTGA	23	60.50	987–1009	
MF3	GAGGCATCTGAGGCCAAGTG	20	60.75	907–926	677
MR3	CCTATTTCCTCATGGATCACTTGC	24	59.48	1560–1583	
MF4	TGGGGRTCATGGGTCATAGCTC	22	61.36	1456–1477	586
MR4	CTGCCCAATCATCAGAAAAGG	22	61.36	2021–2041	
MF5	GGAGTCCGGACTCAAAATGTC	21	58.65	1850–1870	685
MR5	GCGTCATCCACYCGTAGCTC	20	60.59	2515–2534	
MF6	TTAGCTCCCTGCAACCAGGC	20	62.48	2386–2405	634
MR6	CCACAAATTTTGGGACATCCAG	22	58.07	2998–3019	
MF7	GGGATGAGACTGCATTCAGTG	21	58.71	2852–2872	527
MR7	ACACAAAGACCGGCCAACACTTC	21	58.71	3356–3378	
LF1	CGCCCAGATGGACTTG	16	54.13	10–25	414
LR1	CCAATGTTATGGCTCCTA	18	50.76	406–423	
LF2	CCATTGTGGTGGTTGAA	17	51.82	378–394	607
LR2	TTGCTTGTTGCTYCTTG	19	57.12	966–984	
LF3	GGCTATTGACAAAACTCA	16	54.13	10–25	414
LR3	AACCACATTACTGCTTAGRT	18	50.76	406–423	
LF4	TCTTGTTGGAAAAGGCATC	19	53.43	1470–1488	608
LR4	GCATCTTTTGGGGCTTAG	20	59.26	2058–2077	
LF5	GATGGAGGGCTTTGTCTC	18	54.67	2026–2043	645
LR5	TTTCTCAATCAATGTGGC	20	54.93	2651–2670	
LF6	AGTACCATAGGTCCAAACTG	20	54.06	2619–2638	557
LR6	TCATTGCTTCCCTAAACG	20	56.50	3156–3175	
LF7	TCYAGGTCAWCTGATCCGTTTA	22	59.10	3140–3161	491
LR7	TTGGTGRGACGCTGCWAT	20	60.96	3611–3630	
LF8	CACAGGCACTTGGTTAGA	18	54.08	3578–3595	638
LR8	CAGGCTGCTAGTYACACC	20	62.53	4196–4215	
LF9	ACTACAGRGCTCTRGATG	18	49.16	3981–3998	557
LR9	CTGGCTCTTGGAACAAGTCTAT	24	62.15	4514–4537	
LF10	GCACCTAACAGGRAAATT	18	52.01	4462–4479	621
LR10	AATGGAMCCACCCCTAC	19	57.41	5064–5082	
LF11	GGACCATGACTTTATGCC	18	52.70	5025–5042	601
LR11	TCGATTGCTCCAGAGGT	19	58.79	5607–5625	
LF12	GGCGACATCCTTAACCT	17	53.20	5504–5520	497
LR12	TTCCACCTCAGCAGACC	19	59.70	5982–6000	
LF13	CTCAGAGGGRTCRCTTGA	18	52.40	5929–5946	440
LR13	ACAAAGACCGCCCAGAT	19	60.00	6350–6368	

SFTS, Severe fever with thrombocytopenia syndrome; Tm, primer melting temperature

### SFTSV whole-genome sequencing and analysis

The PCR products were verified by agarose gel electrophoresis and sent to Sangon Biotech (Shanghai) Co., Ltd. for sequencing. Sequences were assembled using the SeqMan program of DNASTAR 7.0.

Full-length sequences of 49 SFTSV isolates from China, Japan and the Republic of Korea, collected from 2010 to 2016, were downloaded from GenBank; these isolates cover the six known (A‒F) genotypes of SFTSV. MEGA6.0 software was used to conduct a comprehensive analysis of the SFTSV strain, with the aim to identify the genotype of the virus and analyze the genetic evolution of the strain. The maximum likelihood (ML) method was used for multiple alignments to build a phylogenetic tree based on the L, M and S sequences. A bootstrap method with 1000 replications was used to estimate the reliability of the results.

## Results

### Tick species identification

The* 16S* rRNA sequences of 59 samples taken for verification purposes had 89.33–100% identity with those of *H. longicornis* (Additional file 1: Dataset S1), and were found to be 87.56–100% identical. Based on PCR–RFLP analysis of the* 16S* rRNA gene, 389 tick samples were identified as *H. longicornis*, and three tick samples [P(7), P(65), P(a96)] were identified as *Haemaphysalis juxtakochi*.

### SFTSV nucleic acid detection

Of the 17 patients with suspected SFTS, five (2 men, 3 women) were confirmed to be infected with SFTSV through molecular diagnostic tests. Of the 492 tick samples, 12 samples were positive for SFTSV nucleic acids, with a positivity rate of 2.4%.

### Virus isolation results

Four SFTSV strains were successfully isolated from two positive patients and two tick samples [P(36) and P(47)]; these were designated LNHDD2019-9 (Dandong City, Liaoning Province), LNHDG2019-6 (Donggang City, Liaoning Province), LNTEDG2019-6 (Erdaogou, Dandong City, Liaoning Province) and LNTYJ2019-6 (Yanji City, Jilin Province) based on the geographic location of the sample source (Fig. 1).

### Systematic evolutionary analysis of the SFTSV strain

The L, M and S fragments of the four SFTSV strains were amplified separately and assembled. The sequences of S, M and L fragments of SFTSV (LNHDD2019-9/Human/China/2019 in the evolutionary tree) were uploaded to the NCBI database with accession numbers MT232960, MT232961 and and MT232962, respectively.

**Fig. 2:**
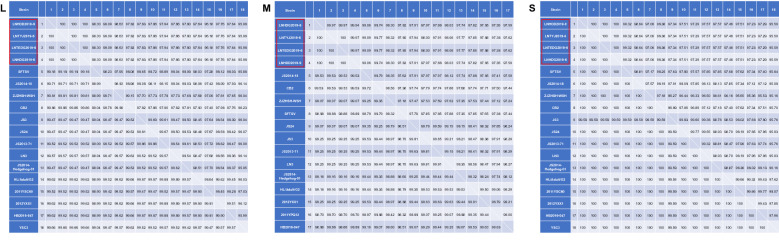
Comparison (% similarity) of nucleotide (top right) and amino acid (bottom left) sequences of L, M and S segments of the SFTSV genotype. The four SFTSV strains successfully isolated in this study are shown in the red boxes

The nucleotide (and deduced amino acid) sequence similarities of the isolates to other genetic A-type strains were 95.95‒100% (98.90‒100%), 97.12‒100% (98.32‒100%), and 95.08‒100% (99.59‒100%) in the L, M and S segments, respectively (Fig. 2), suggesting that the four SFTSV strains isolated are genotype A strains.

**Fig. 3:**
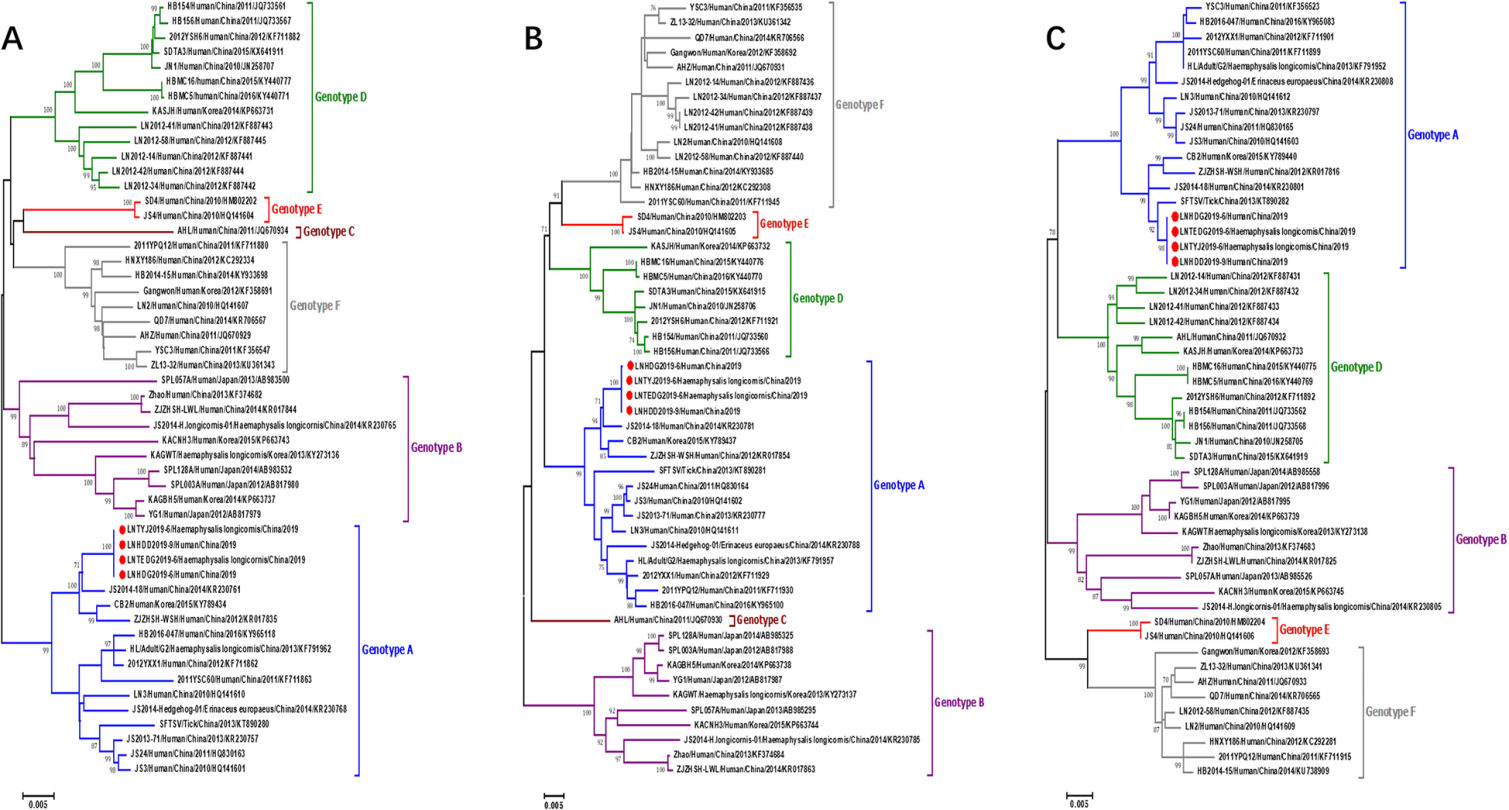
Phylogenetic analyses. Phylogenetic analysis based on the complete nucleotide sequences of the L, M and S segments of the four SFTSV isolates identified (filled red circles). All four isolates are SFTSV genotype A strains.

The amino acid sequences of the M and S segments have been used to distinguish the six SFTSV genotypes [22]. Therefore, we used the ML method for phylogenetic analysis based on amino acid sequences. The results showed that the L, M and S segments of the four SFTSV isolates identified in this study clustered in a clade of genotype A (Fig. 3a–c) and were closest to the viruses isolated in Jiangsu, Zhejiang and other provinces.

The L and S segment sequences of SFTSV isolated from the ticks in Dandong City, Liaoning Province, and Yanji City, Jilin Province, were 100% identical to those of SFTSV strains isolated from patients. In contrast, the similarity of the M segment sequences was 99% (Table 2). These results indicated that the main transmission sources of SFTS in patients were ticks distributed throughout the local or nearby areas.

**Table 2:** Sequence information for SFTSV isolates

SFTSV strain	Source	Geographical origin^a^	Time of isolation	Genotype (L/M/S)^b^	Sequence similarity (%)					
					L segment		M segment		S segment	
					Nucleotide	AA	Nucleotide	AA	Nucleotide	AA
LNHDD2019-9	Human serum	DD, LN	September 2019	A/A/A	99.98	100	99.08	99.63	100	100
LNHDG2019-6	Human serum	DG, LN	June 2019	A/A/A	99.98	100	99.08	99.63	100	100
LNTEDG2019-6	*Haemaphysalis longicornis*	Erdaogou, DD, LN	June 2019	A/A/A	99.98	100	99.08	99.63	100	100
LNTYJ2019-6	*H.longicornis*	YJ, JL	June 2019	A/A/A	99.98	100	99.08	99.63	100	100

AA, Amino acid; SFTSV, severe fever with thrombocytopenia syndrome virus

^a^DD, Dandong City; LN, Liaoning Province; DG, Donggang City; YJ, Yanji City; JL, Jilin Province

^b^The SFTSV genome is composed of three independent gene segments: L segment (6368 bp), M segment (3378 bp) and the antisense S segment (1744 bp). There are 6 known (A‒F) genotypes of SFTSV. For more detail, see text

## Discussion

In this study, the detection, isolation and evolution analysis of SFTSV isolates were carried out using serum collected from patients suspected of having SFTS and from ticks in the area surrounding their homes. The results show that five of 14 patients with suspectged SFTS and 12 of 492 ticks were positive for SFTSV. Whole-genome sequencing of the four isolated strains of SFTSV revealed that all were SFTSV genotype A. This is the first report of genotyping on the SFTSV genotype in Liaoning Province, and the findings have deepened our understanding of the SFTS epidemic.

To the best of our knowledge, this is the first work identifying SFTSV genotypes in patients with SFTS and ticks in Liaoning Province. Previous studies in Liaoning have focused on epidemiological characteristics and symptoms and mixed symptoms of SFTS. The SFTSV strains isolated in this study were all genotype A, consistent with previous reports that the dominant strain of SFTSV in China is genotype A.

Northeast China is characterized by a mountainous terrain with abundant natural resources, geographical complexity and significant biodiversity, all factors that provide an ecological and biological basis for the survival and reproduction of ticks. The tick species identified in this study are *H. longicornis*,* Dermacentor silvarum*,* D. nuttalli* and* Ixodes persulcatus* [19]. In a previous study, *H. longicornis* was the dominant species collected in northeast China and the only tick species collected in Liaoning province [19]. Our results showing that most of the ticks collected in the present study were* H. longicornis* verifies this earlier finding. However, three samples were confirmed to be* H. juxtakochi* by PCR–RFLP analysis of the* 16S* rRNA gene. This tick species has not been found in China earlier. Further investigation and verification are required in future studies.

In this study, the SFTSV-positive rate of ticks was 2.4%, which is lower than that (3.0%) reported in Jilin province [19]. However, the number of SFTS cases (708) in Liaoning Province in 2010–2019 was much higher than that (< 94) in Jilin Province [23], which may be related to the activities (working and social) of the people in the area, their awareness of potential illness associated with ticks and testing methods.

Reports from the East Asian region show that the average mortality rate of SFTSV varies greatly, from 5.3 to 16.2% in China [9] to 20% in Japan [24] and 23.3% in South Korea [25]; in comparison, mortality was 5.3% in 2011–2014 [9] and 3.2% in 2010–2019 [23] in Liaoning Province. Among the six known A-F genotypes of SFTSV [26], genotype B is associted with the highest morbidity and mortality rates. In one study, the incidence of the F genotype was lower than that of the B genotype, but the associated mortality rate of the former was higher, while genotype A had the lowest mortality rate [26]. The patients with SFTS in this study were infected from the end of March to the end of June. They all developed the most frequent symptoms of SFTS, such as fever, headache, dizziness, muscle joint pain, nausea and vomiting. After treatment, they all recovered. The identification of SFTSV genotype A in the present study explains the low mortality rate of patients with SFTS in Liaoning Province.

The most common genotypes of SFTSV in China are genotypes A, D and F, which are associated with relatively low mortality rates. In contrast, the most common genotype of SFTSV in South Korea is genotype B, with a high mortality rate [22, 26, 27]. Because of the geographical proximity between Liaoning and South Korea, genotype B may soon spread to China. Therefore, it is important to improve methods for detecting SFTSV genotypes to control and diagnose highly lethal SFTS.

In Liaoning Province,* H. longicornis* is the dominant tick species; the other species identified include * Haemaphysalis concinna*,* H. japonica* and* I. persulcatus*. In the present study, only* H. longicornis*-infected ticks were identified. Further studies are required to identify different tick species carrying other SFTSV. At the same time, this study only collected ticks in counties and cities of the patients. Tick species in other counties and towns and the SFTSV infection pattern must be further investigated.

## Conclusion

The study identified SFTSV genotypes in patients with suspected SFTS and in ticks in Liaoning Province. The results enhance our understanding of the SFTS epidemic in Liaoning Province. Because the highly lethal genotype B is the main epidemic strain in South Korea, it is critical to improve methods for detecting SFTSV genotypes to prevent the occurrence and spread of highly lethal SFTSV.

## Supplementary Information

**Additional file 1: 16S.mas.** The alignment of the* 16S* rRNA sequences of 59 tick samples.

## Data Availability

The datasets supporting the conclusions of this article are included within the article.

## Abbreviations

CK: Creatine kinase
ML: Maximum likelihood
SFTS: Severe fever with thrombocytopenia syndrome
SFTSV: SFTS virus
PBS: Phosphate-buffered saline
RFLP: Restriction fragment length polymorphism
